# Tyrosine kinase inhibitors were well-tolerated among patients with different etiologies of advanced HCC with lower survival in non-viral patients

**DOI:** 10.1038/s41598-025-05828-x

**Published:** 2025-06-27

**Authors:** Eman M. F. Barakat, Mohamed Kohla, Hossam Dabees, Hend Ibrahim Shousha, Ehab F. Moustafa, Mohamed El-Kassas, Mona Shoukry Aziz, Eman Elkhateeb, Ashraf Omar Abdelaziz, Mohamed Omar Abdelmalek, Aly Azmy, Ahmed Tawheed, Walaa Mosaad Aboganob, Hossam Taha, Rania Lithy, Ahmed Radwan, Dalia Ghoraba, Hamdy Sayed, Anwar Nassief, Mostafa Elhelbawy, Mohamed Mahmoud nabeel, Mohamed A. Medhat, Safaa Ragab Askar, Eman Marwan, Eman Rewisha, Tamer Elbaz, Sayed Hassan Ahmed, Nevien Fouad elfouly, Nermeen Abdeen, Ahmed Hosni Abdelmaksoud, Asmaa A. Abdeltawab, Mostafa Abd Alfattah Shamkh, Ahmed Ramadan, Yasser Arafat Abdelrazek, Mohamed Bassam, Sayed Ahmed Sayed, Rasha Salah Hussein, Ammar Alrajhi, Allam Elsayed Allam, Omnia A. Seyam, Mohamed Said

**Affiliations:** 1 Hepatoma Group, Tropical Medicine Department, Ain Shams University, Cairo, Egypt; 2 Hepatology and Gastroenterology Department, National Liver Institute, Menoufia University, Menoufia, Egypt; 3 National Medical Institute of Damnhour, Damnhour, Egypt; 4 Endemic Medicine and Hepato-Gastroenterology Department, Cairo University, Cairo, Egypt; 5 Department of Tropical Medicine and Gastroenterology, Assuit University, Assuit, Egypt; 6 Endemic Medicine Department, Faculty of Medicine, Helwan University, Cairo, Egypt; 7 Department of Medical Oncology and Nuclear Medicine, Ain Shams University, Cairo, Egypt; 8 School of Medicine, Newgiza University, Giza, Egypt; 9 Diagnostic and Interventional Radiology, Faculty of Medicine, Assuit University, Assuit, Egypt; 10 National Center for Radiation Research and Technology Egyptian Atomic Energy Authority, Cairo, Egypt; 11 Tropical Medicine Department, Alexandria University, Alexandria, Egypt; 12 Diagnostic and Interventional Radiology Department, Cairo University, Cairo, Egypt; 13 Oncology Department, Faculty of Medicine, Assuit University, Assuit, Egypt; 14 Diagnostic and Interventional Radiology Department, Ain Shams University, Cairo, Egypt

**Keywords:** Hepatocellular carcinoma, Sorafenib, Regorafenib, Hepatitis, Survival, Hepatology, Hepatocellular carcinoma

## Abstract

We studied the characteristics and survival of patients with sorafenib-treated HCC and impact of underlying etiology on outcomes. This retrospective multicenter study recruited patients with sorafenib-treated advanced HCC (12/2016 to 4/2023) till death or the study end (2/2024). Time to progression (TTP) and overall survival (OS) were recorded. We evaluated; Clinico-laboratory and imaging predictors of OS, The impact of underlying etiology on tumor variables, outcomes and tolerance for sorafenib > 6 months. This study included 706 patients. Median duration of Sorafenib therapy was 240.00 (90.00–360.00) days. Median OS was 314.00(146.00–601.00) days. Median TTP was 180.00(90.00–330.00) days. COX regression revealed that the independent factors of mortality were baseline AST, Tumor size, hepatic vein thrombosis (HVT), development of jaundice and shifting to Regorafenib. Advanced HCCs were more common on top of non-cirrhotic non-viral and HBV-related liver disease. Adverse events, TTP and tumor response didn’t differ with the underlying etiology. Median OS was lower in non-viral-related HCC than HCV-related HCC (218.00 versus 326.50 days, *P*-value = 0.048). Patients who continued sorafenib > 6 months had lower AFP, HVT, adverse effects and better tumor response after 3 months. OS is lower in non-viral Sorafenib-treated HCC compared with viral-related HCC and Sorafenib was well-tolerated among different HCC etiologies.

## Introduction

Globally, hepatocellular carcinoma (HCC) is ranked as the third most common contributor to cancer-related mortality and the sixth most common cancer^1^. HCC can be triggered by chronic hepatitis B or C viral infections, alcohol misuse, and metabolic syndrome. Liver cirrhosis, which affects 85–95% of HCC patients, is the main risk factor^2^. The majority of HCC patients receive their diagnosis too late, missing the best window of opportunity for surgery^3,4^.

Since 2008^5^, Sorafenib, a multi-targeted tyrosine kinase inhibitor (TKI), has been the accepted line of treatment for advanced HCC. Regorafenib, on the other hand, is authorized as a second chance for non-responders to sorafenib. When compared to the placebo in the RESORCE trial, Regorafenib dramatically increased both OS and progression-free survival^6^. Systemic therapies for HCC have progressed to the point where Lenvatinib and, later, immunotherapy combinations of Atezolizumab plus Bevacizumab have been approved as first-line treatments. As a second-line treatment option, Cabozantinib, Ramucirumab, immune checkpoint inhibitors (ICIs) Pembrolizumab, and Nivolumab plus Ipilimumab are now offered^7^.

Although sorafenib has been successfully used to treat advanced HCC, essential predictors of its effectiveness remain to be determined^8^. Sorafenib-related side effects include diarrhea, hypertension, and hand-foot syndrome (HFS)^5^. Even though some side effects can predict effectiveness or signal the need for dose modification, there are no established guidelines to adhere to^9^. Even though Regorafenib’s overall safety profiles were comparable to those of sorafenib and its representative adverse effects were rare, 25% of patients in the RESORCE trial stopped receiving therapy as a result of the drug’s side effects^6^. These results are unexpected and reveal differences in the mechanisms underpinning the negative effects generated by Sorafenib and Regorafenib, as well as the mechanisms governing their anti-tumor actions, despite the very similar chemical structures of the two drugs^6^. While comprehensive data was required to examine this clinical query, no report offered a comparison of the two medications’ individual levels of safety and efficacy.

This research includes, for the first time, a large Egyptian population that received Sorafinib and the second line, Regorafenib. There is never enough information available from clinical trials about how patients with HCC from the Middle East and North Africa (MENA) area react to such medications. There is conflicting evidence about the impact of underlying etiology on sorafinib response. The SHARP study, which resulted in the approval of sorafenib, did not conduct thorough etiology-based subgroup analysis^10^, and the findings of further research have been inconclusive. While previous real-world trials indicate modestly better survival in patients with viral-related HCC^11,12^, Hiraoka et al.^13^ found no significant differences in sorafenib effectiveness across HBV, HCV, and non-viral subgroups.

Regional and ethnic differences may affect adverse events pattern and OS as reported by the GIDEON trial^14^. For example, compared to Western populations, patients from the Asia–Pacific area, who were primarily of East Asian origin, tended to have a worse OS. This might be due to variations in liver function, treatment accessibility, and disease stage upon diagnosis. Similarly, despite similar disease control rates, a sub-analysis of the SHARP and Asia–Pacific trials showed that the median OS in the Asia–Pacific cohort was 6.5 months, which was much shorter than the 10.7 months seen in the SHARP trial (Western population)^9,15^. Due to pharmacogenomic variations in sorafenib metabolism or drug sensitivity, Asian patients seem to experience greater rates of certain toxicities, such as diarrhea and hand-foot skin reaction^16,17^. On the other hand, fatigue and hypertension are more common in Western patients.

In addition to examining the effects of the patients’ underlying disease etiology on treatment success, this work seeks to evaluate the characteristics, adverse events, treatment duration and outcomes, and OS of patients with sorafenib-treated advanced HCC.

## Patients and methods

This study is a retrospective multicenter effort. Six tertiary care centers provided patients for the treatment of HCC:

1. Viral hepatitis center, National medical institute of Damanhur, Boheira Governorate
2. National Liver Institute, Menoufia University, Menoufia Governorate
3. Multidisciplinary HCC clinic, Cairo University, Cairo Governorate
4. Assuit Hepatoma Group, Assuit University, Assuit Governorate
5. Hepatoma group, Tropical Medicine Department, Ain-Shams University, Cairo Governorate
6. Endemic medicine department, Helwan University, Cairo Governorate

All Sorafenib-treated Patients were recruited during the study period from December 2016 to April 2023, and follow-up continued till death or the end of the study in February 2024. No particular grant from a public, private, or nonprofit organization has been provided for this research. This study adhered to the World Medical Association’s 1975 Code of Ethics (Declaration of Helsinki) and its subsequent revisions. An informed consent was obtained from all participants of the study. The protocol was approved by the ethics committees of Cairo University’s Faculty of Medicine (number: N-336-2023), Ain-Shams University’s Faculty of Medicine (number: FMASU-R36-2024), and Assiut University’s Faculty of Medicine (number: 04-2023-300142).For every patient, we gathered baseline demographic information, ECOG performance status, Child–Pugh Score and laboratory investigations (Complete blood count (CBC), hepatic and renal function tests, HBsAg, Anti-HCV Ab and Alpha-fetoprotein (AFP)). As well, Echocardiogram (ECG) was done, and if needed Echocardiography.

In compliance with globally accepted recommendations, the diagnosis of HCC was verified using either a Triphasic CT or a dynamic MRI^18^. The Barcelona Clinic Liver Cancer (BCLC) staging was performed upon diagnosis. We included patients with BCLC B or C. Patients then started sorafenib therapy (200 mg once daily, increased to 200 mg twice daily, then if the previous dose is tolerated increase to 400 mg BID). Tumor response was evaluated using the modified Response Evaluation Criteria in Solid Tumors (modified RECIST)^19^.

### Follow-up

The initial evaluation of response was performed after 1 month by Triphasic CT or dynamic MRI, or PET CT if needed, then every 3 months. Also, patients were assessed for compliance with treatment and the development of adverse events (AEs). The severity of AEs was evaluated if necessitating dose modification, stopping treatment, or shifting to other drugs. Follow-up was performed until a patient’s death or until the end of the study for surviving patients**.**

Follow-up laboratory investigations including CBC, hepatic and renal function tests, AFP, after 1 month and then every 3 months. In addition, an ECG was done every month for every patient. Regorafenib was the available second-line therapy for patients with progressive disease (160 mg PO daily for the first 21 days of each 28-day cycle). Regorafenib-related AEs, follow-up laboratory data, and the modified RECISET criteria after Regorafenib were recorded. Time to progression (TTP) was calculated from the date of treatment start until the occurrence of tumor progression. Overall survival (OS) was calculated from the date of treatment start till patient death or the study end.

Due to the conflicting results reported about the effect of underlying liver disease etiology on the outcome of Sorafenib treatment, we divided the patients into hepatitis C-related HCC, Hepatitis-B related HCC and non-viral HCC to examine the effects of the underlying disease etiology on patients characteristics, adverse events, treatment duration and outcomes, and OS. The primary endpoint of the study was the overall survival (OS) of the recruited patients and time to progression (TTP), treatment related adverse events. The Secondary endpoints: effect of duration of treatment on treatment outcome.

### Statistical methods

The statistical software for the social sciences (SPSS) version 28 (IBM Corp., Armonk, NY, USA) was utilised to code and enter the data. For quantitative variables, the data was summarized using the median and interquartile range; for categorical variables, the data was summarized using frequencies (number of cases) and relative frequencies (percentages). Non-parametric Mann–Whitney and Kruskal–Wallis tests were utilised to compare quantitative variables. We used the Chi-square (2) test to compare categorical data. When the expected frequency is less than five, an exact test was utilized in its place. The Spearman correlation coefficient was utilized to perform correlations between quantitative variables. In both univariate and multivariate regression models, the Cox proportional hazards were used to evaluate independent prognostic variables. *P*-values were regarded as statistically significant if they were *P*  < 0.05.

## Results

This multicenter retrospective work included 706 consecutive patients with Sorafenib-treated HCC. The median age of the studied patients was 62 years with male predominance (76.1%), 98% of our patients had cirrhosis, with the majority had Child–Pugh class A (96.5%) (Table 1). Regarding tumor characteristics, 55.5% had a single focal lesion and 27.3% had main portal vein thrombosis, 59.5% had BCLC stage-B (Table 2). The median (IQR) duration of Sorafenib treatment was 240.00 (90.00–360.00) days. The most common causes of treatment discontinuation were Liver decompensation (26.3%) followed by patient demand (16.4%). On the other hand, 177 (25.1%) patients are still on treatment (Table 3).

**Table 1:** Demographic and laboratory features of the studied patients.

Variables median (IQR)		Patients with HCC treated with sorafenib	
		Count	%
Age (years) (median and IQR)		62.00	57.00–67.00
Gender	Male	537	76.1%
	Female	169	23.9%
Cigarettes Smoking		110	15.6%
Chronic medical illness	Systemic hypertension	42	5.9%
	Diabetes mellitus	161	22.8%
	COPD	1	0.1%
	Bronchial asthma	1	0.1%
Liver cirrhosis		692	98.0%
Splenomegaly		579	82.0%
Antiviral intake		540	76.5%
Responders to antivirals		512	72.5%
Ascites	No	608	86.1%
	Minimal	27	3.8%
	Mild	71	10.1%
ECOG Performance status	0	487	69.0%
	1	178	25.2%
	2	41	5.8%
Child–Pugh Score	A	681	96.5%
	B	25	3.5%
Hemoglobin (gm/dl)		12.50 (11.20–13.60)	
Total leucocyte count (× 10^3^/ul)		6.00 (4.40–7.80)	
Platelets (× 10^3^/ul)		165.00 (116.00–224.00)	
Total bilirubin (mg/dl)		0.90 (0.70–1.20)	
Alanine transferase (ALT) (U/L)		35.00 (24.00–49.00)	
Aspartate transferase (AST) (U/L)		45.00 (32.00–70.00)	
Serum albumin (gm/dl)		3.70 (3.50–4.00)	
Creatinine (mg/dl)		0.96 (0.80–1.10)	
International normalized ratio		1.10 (1.03–1.23)	
Alpha-fetoprotein (U/L)		255.50 (22.00–1210.00)	
Hepatitis B surface antigen Count (%)		15	2.1%
Hepatitis C Antibodies Count (%)		622	88.1%

**Table 2:** HCC Tumor characteristics of the studied patients.

Variables		Patients with HCC treated with sorafenib	
		Count	%
Tumor size (median (IQR)) in cm		7.00 (5.00–10.00)	
Number of Focal lesions	Single	392	55.5%
	Two	104	14.7%
	Three	34	4.8%
	Multiple	176	24.9%
Tumor site	Right lobe	508	72.0%
	Left lobe	86	12.2%
	Both lobes	112	15.9%
Portal vein status	Patent	469	66.4%
	Segmental	1	0.1%
	Thrombosed main portal vein	193	27.3%
	Right PVT	30	4.2%
	Left PVT	9	1.3%
	Right and Left PVT	3	0.4%
	SMV thrombosis	1	0.1%
Malignant lymphadenopathy		188	26.6%
hepatic vein thrombosis		31	4.4%
Extra hepatic spread	Bone	6	0.8%
	Bone, Suprarenal, Peritoneal	1	0.1%
	Lung	18	2.5%
	Lung and Bone	7	1.0%
	Suprarenal	2	0.3%
	no	672	95.2%
BCLC staging	B	420	59.5%
	C	286	40.5%

**Table 3:** Treatment with sorafenib and its outcome.

Variable			Count	Percent
Previous treatment before starting sorafenib			137	19.4%
Duration of Sorafenib intake median (IQR) (in days)	240.00 (90.00–360.00)			
Median Survival duration (IQR) (in days)	314.00 (146.00–601.00)			
Median Time to progression (IQR) (days)	180.00 (90.00- 330.00)			
Died			379	53.7%
The modified RECIST criteria after Sorafenib	Stable disease		219	45.63%
	Progressive disease		161	33.54%
	Partial response		100	20.83%
Causes of Sorafenib discontinuation	Liver decompensation		186	26.3%
	Patient demand		116	16.4%
	Died		50	7.1%
	Progressive course of HCC		42	5.9%
	Anemia		22	3.1%
	Diarrhea		17	2.4%
	Dermatological complications		12	1.7%
	Elevated liver enzymes		9	1.3%
	Renal impairment		7	1.0%
	Fatigue		7	0.9%
	Abdominal pain		5	0.7%
	Hypertension		3	0.4%
	Persistent vomiting		2	0.3%
	Arrhythmia		1	0.1%
	Developed gastric cancer		1	0.1%
	Disturbed conscious level		1	0.1%
	Fever and abdominal pain		1	0.1%
	Hypothyroidism		1	0.1%
	Ischemic cardiomyopathy		1	0.1%
	Massive hematemesis		1	0.1%
	Pancytopenia		1	0.1%
	Peripheral neuropathy		1	0.1%
	Recurrent severe hypoglycemia		1	0.1%
	Severe anorexia		1	0.1%
	Squint		1	0.1%
	Sub-acute DVT		1	0.1%
Shifted to Regorafenib			68	9.6%
The modified RECIST criteria after Regorafenib	Stable disease		27	43.5%
	Progressive disease		18	29.0%
	Partial response		17	27.4%
Causes of Regorafenib discontinuation	Liver decompensation		43	64.2%
	Progressive disease		18	26.9%
	Life threatening arrhythmia		2	3.0%
	Renal tubular acidosis		1	1.5%
	Fatigue		1	1.5%
	Bleeding skin ulcer		1	1.5%
	Acute Myocardial infarction		1	1.5%
Adverse events of Regorafenib	Hypertension		11	1.6%
	Diarrhea		7	1.0%
	Hand and foot syndrome, perineal ulcers		2	0.3%
	Skin ulcers		1	0.1%
	Liver decompensation		43	64.2%
	Hand and foot syndrome		1	0.1%
	Generalized itching, fatigue, loss of appetite		1	0.1%
	Anemia, RTA		1	0.1%
	Life threatening arrhythmia		2	0.2%
	Acute Myocardial infarction		1	0.1%
	ALT 1 month after Regorafenib	Grade 1	25	96.2%
		Grade 2	1	3.8%
	AST 1 month after Regorafenib	Grade 1	24	88.9%
		Grade 2	3	11.1%
	ALT 3 months after Regorafenib	Grade 1	27	96.4%
		Grade 2	1	3.6%
	AST 3 months after Regorafenib	Grade 1	34	97.1%
		Grade 2	1	2.9%

ALT, AST: grade 1 (> ULN to 3 folds), Grade 2 (> 3.0—5.0 folds), Grade 3 (> 5.0—20.0 folds), Grade 4 (> 20.0 folds).

**Fig. 1:**
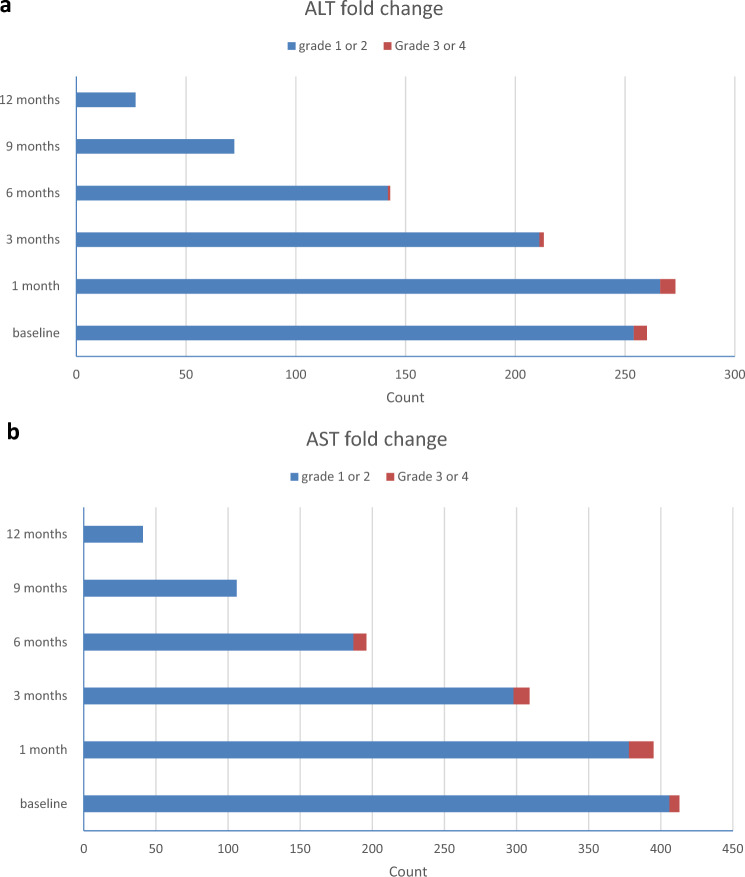
(**a**) Fold elevation in ALT during sorafenib therapy (**b**) Fold elevation in AST during sorafenib therapy.

*Sorafenib-related adverse events* are reported in Table 4. Jaundice is the most commonly reported adverse event (30.9%) followed by fatigue (24.6%), anemia (19.3%), and elevated liver enzymes (15.4%) (Fig. 1). None of the patients who continued sorafenib treatment for more than 6 months developed grade 3 or 4 elevations of liver enzymes. Changes in laboratory parameters and ECOG performance status are shown in Supplementary Tables 1, 2

**Table 4:** Sorafenib-related adverse events.

		Count	%
Hypertension		87	12.3
Hypertension Grade	1	64	9.1
	2	18	2.5
	3	5	0.7
Hand and foot syndrome		31	4.4
Hand and foot syndrome Grade	1	16	2.3
	2	10	1.4
	3	5	0.7
Skin lesions		92	13.0
Skin lesions Grade	1	63	8.9
	2	25	3.5
	3	4	0.6
Diarrhea		56	7.9
Diarrhea Grade	1	45	6.4
	2	10	1.4
	3	1	0.1
Fatigue		174	24.6
Fatigue Grade	1	146	20.7
	2	22	3.1
	3	6	0.8
Abdominal pain		76	10.8
Abdominal pain Grade	1	66	9.3
	2	5	0.7
	3	3	0.4
	4	2	0.3
Jaundice		218	30.9
Jaundice Grade	1	116	16.4
	2	82	11.6
	3	20	2.8
Anemia		136	19.3
Elevated liver enzymes		109	15.4
Ascites		47	6.7
Nausea/vomiting		27	3.8
Hepatic encephalopathy		26	3.7
Hematemesis		14	2.0
Other side effects	Renal impairment	14	1.9
	DCL	8	1.1
	Anorexia	6	0.8
	Myocardial ischemia	5	0.7
	Hypothyroidism	4	0.6
	Fever	3	0.4
	Arrhythmia	2	0.2
	Dysphonia (vocal cord edema)	2	0.2
	Alopecia	2	0.3
	Hoarseness of voice	2	0.2
	Leucopenia	2	0.3
	Peripheral neuropathy	2	0.2
	Altered taste	1	0.1
	Bleeding gums	1	0.1
	Chest tightness	1	0.1
	Glossitis	1	0.1
	Hair loss and whitening	1	0.1
	Melena	1	0.1
	Myocardial infarction	1	0.1
	Pancytopenia	1	0.1
	Recurrent hypoglycemia	1	0.1
	Severe bone pain	1	0.1%
	Steven Johnson Syndrome	1	0.1
	Subacute DVT	1	0.1

The modified RECIST criteria were available for 480 (68%) of patients. None of the patients achieved a complete response. One hundred (20.83%) patients achieved partial response. On the other hand, 219 (45.63%) patients had a stable disease and 161 (33.54%) patients showed progressive disease (Table 3).

By the end of the study, 379 (53.7%) patients died. The median (IQR) OS after Sorafenib treatment was 314.00 (146.00–601.00) days (10.47 months (4.87–20.03). The median (IQR) time to progression was 180.00 (90.00- 330.00) days (Table 3). We found no difference in the median OS between patients below 65 years-old and elderly patients above 65 years old (314.00 (139.00- 612.00) and 314.50 (154.00- 594.00) respectively, *P*-value 0.986). In addition, we found no difference in the median OS between patients with Child–Pugh A and B (310.50 (143.00–601.00) and 373.50 (228.00–608.00) days, respectively, *P*-value 0.529) or between patients with BCLC B and C (307.00 (141.00–594.00) and 334.00 (154.00–608.00) days, respectively, *P*-value 0.406).

Spearman correlation coefficient revealed a strong positive correlation between the duration of treatment with sorafenib and the median survival time (r = 0.732, *P*-value < 0.001) and a negative correlation between the duration of sorafenib treatment and baseline AFP (r = − 0.202, *P*-value < 0.001) (Supplementary Table 3). Comparison between baseline laboratory data and data before treatment stop revealed significant reduction in blood counts and deterioration of liver function tests with no significant changes in serum Creatinine or AFP (Supplementary Table 4).

Univariate *COX regression analysis* revealed that factors affecting survival after Sorafenib treatment are cigarette smoking, performance status, the presence of splenomegaly, serum bilirubin and AST, in addition to the number, site and size of the hepatic focal lesions, the presence of hepatic vein thrombosis, the development of fatigue, and shifting to Regorafenib (Table 5). *Multivariate regression analysis* revealed that the independent factors affecting mortality were baseline serum AST (*P* value < 0.001, Hazard ratio (HR) 1.005, 95.0% CI (1.002–1.007)), size of HCC (*P* value 0.007, HR 1.039, 95.0% CI (1.010–1.068)), the presence of hepatic vein thrombosis before treatment carries 1.739 times higher risk of mortality (*P* value 0.013, HR 1.739, 95.0% CI (1.125–2.688)), the development of jaundice during sorafenib treatment carries 2.192 times higher mortality risk (*P* value < 0.001, HR 2.192, 95.0% CI (1.756–2.737)), on the other hand shifting to Regorafenib is associated with 0.340 lower risk of mortality (*P* value < 0.001, Hazard ratio (HR) 0.340, 95.0% CI (0.205–0.566)).

**Table 5:** Univariate COX regression analysis for factors affecting survival.

		*P* value	Hazard ratio	95.0% Confidence interval	
				Lower	Upper
Age (years)		0.601	1.003	0.991	1.016
Hemoglobin (gm/dl)		0.223	0.964	0.908	1.023
Total leucocyte count (× 10^3^/ul)		0.652	1.008	0.973	1.044
Total bilirubin (mg/dl)		0.038	1.271	1.013	1.594
Alanine transferase (ALT) (U/L)		0.720	1.001	0.998	1.003
Aspartate transferase (AST) (U/L)		< 0.001	1.005	1.003	1.008
Serum albumin (gm/dl)		0.402	1.094	0.886	1.352
Creatinine (mg/dl)		0.129	1.254	0.936	1.681
International normalized ratio		0.496	0.817	0.456	1.463
Alpha-fetoprotein (U/L)		0.055	1.000	1.000	1.000
F.L size or size of largest lesion if 2 or multiple		0.005	1.040	1.012	1.069
Gender	Male	0.268	1.155	0.895	1.491
Smoker		< 0.001	0.385	0.264	0.561
Chronic medical illness		0.914	1.013	0.803	1.277
Performance status	1	0.206	0.853	0.667	1.091
	2	0.206	1.351	0.847	2.156
Liver	Cirrhosis	0.932	1.033	0.489	2.183
Spleen status	Splenomegaly	0.031	1.384	1.029	1.860
	Surgically removed	0.448	1.570	0.490	5.031
Ascites	Minimal	0.253	0.692	0.369	1.301
	Mild	0.144	1.270	0.922	1.749
CHILD score	B	0.885	0.960	0.551	1.672
Number of focal lesions	2	0.052	0.738	0.542	1.003
	3	0.042	1.552	1.016	2.369
	Multiple	0.001	0.636	0.488	0.831
F.L site	Right lobe	0.001	1.668	1.223	2.274
	Left lobe	0.678	0.912	0.590	1.410
Portal vein status	Thrombosed	0.314	0.892	0.714	1.114
Hepatic vein thrombosis		0.002	1.942	1.270	2.969
Extra hepatic spread		0.186	0.701	0.414	1.187
BCLC	C	0.809	0.974	0.787	1.206
Previous treatment before Sorafenib		0.057	0.768	0.584	1.008
Sorafenib interrupted or not		0.739	1.058	0.759	1.476
Hypertension		0.857	1.029	0.751	1.411
Hand and foot syndrome		0.148	0.653	0.367	1.163
Skin lesions		0.950	0.990	0.734	1.337
Diarrhea		0.417	0.852	0.579	1.254
Fatigue		0.009	0.720	0.563	0.921
Abdominal pain		0.982	0.996	0.719	1.380
Jaundice		< 0.001	2.531	2.042	3.138
Elevated liver enzymes		0.472	1.108	0.838	1.466
Hematemesis		0.434	1.305	0.670	2.544
Nausea/vomiting		0.053	0.520	0.268	1.009
Shifted to Regorafenib		< 0.001	0.276	0.167	0.456
Hepatitis C Antibodies		0.060	0.729	0.524	1.014
Hepatitis B surface antigen		0.339	1.402	0.701	2.805

Regarding Regorafenib therapy, Sixty-eight (9.6%) patients were shifted to Regorafenib as a second-line therapy. None of the patients achieved complete response while 17 (27.4%) patients achieved partial response. The most prevalent adverse event was mild elevation of liver enzymes followed by hepatic decompensation and then hypertension. The most common cause of Regorafenib discontinuation was hepatic decompensation (64.2%) (Table 3).

We divided our patients according to the etiology of their underlying chronic liver disease into 3 groups: 622 (88.1%) patients with HCV-related HCC, 15 (2.1%) patients with HBV-related HCC, and 69 (9.8%) patients with non-viral-related HCC. Patients with HBV-related HCC were significantly younger with significantly lower baseline total leucocytes count. Baseline ALT was significantly lower in patients with HCV-related HCC. Baseline AST was significantly higher in patients with HBV-related HCC. These advanced unresectable HCC lesions developed on top of non-cirrhotic liver in 7.1% of patients with HBV and 7.1% of patients with non-viral etiology. On the other hand, it developed on top of 1.3% of patients with non-cirrhotic HCV infection. Extra-hepatic spread was significantly more common in patients with HBV-related HCC (Table 6).

**Table 6:** Features of the studied patients with HCC according to their underlying etiology.

		Chronic hepatitis C (N = 622)		Chronic hepatitis B (N = 14)		Non-viral (N = 70)		*P* value
		Count	%	Count	%	Count	%	
Age (years)		62.00 (57.00–67.00)^a^		57.00 (48.00–59.00)^b^		65.00 (60.00–70.00)^a^		< 0.001
Gender	Male	471	75.7%	11	78.6%	55	78.6%	0.916
	Female	151	24.3%	3	21.4%	15	21.4%	
Cigarette Smoking		98	15.8%	4	28.6%	8	11.4%	0.246
Chronic medical illness		185	29.7%	3	21.4%	17	8.6%	0.609
Performance status	0	431	69.3%	11	78.6%	45	64.3%	0.076
	1	159	25.6%	2	14.3%	17	24.3%	
	2	32	5.1%	1	7.1%	8	11.5%	
Antiviral therapy		535	86.0%	5	35.7%	0	0.0%	–
Responders to antivirals		512	82.3%	0	0.0%	0	0.0%	–
Liver status	Cirrhosis	614	98.7%	13	92.9%	65	92.9%	0.005
	Non-cirrhosis	8	1.3%	1	7.1%	5	7.1%	
Spleen	Enlarged	505	81.2%	11	78.6%	63	90.0%	0.374
	Surgically removed	5	0.8%	0	0.0%	0	0.0%	
	Average	112	18.0%	3	21.4%	7	10.0%	
Ascites	Minimal	22	3.5%	2	14.3%	3	4.3%	0.238
	Mild	63	10.1%	2	14.3%	6	8.6%	
	No	537	86.3%	10	71.4%	61	87.1%	
Hemoglobin (gm/dl)		12.50(11.20–13.60)		12.35 (12.00–14.30)		12.00 (11.10–13.40)		0.301
Total leucocyte count (× 10^3^/ul)		6.00 (4.30–7.90)^a^		4.15 (3.40–5.00)^b^		6.30 (4.80–8.10)^a^		0.009
Platelets (× 10^3^/ul)		164.00 (112.00–224.00)		148.00 (115.00–179.00)		181.00 (131.00–230.00)		0.284
Total bilirubin (mg/dl)		0.90 (0.70–1.20)		1.00 (0.80–1.10)		0.90 (0.60–1.20)		0.803
Alanine transferase (ALT) (U/L)		33.15 (24.00–48.00)^b^		44.00 (40.00–60.00)^a^		40.00 (28.00–61.00)^a^		0.002
Aspartate transferase (AST) (U/L)		45.00 (32.00–67.00)^a^		62.00 (46.00–116.00)^b^		50.10 (34.00–77.00)^ab^		0.017
Serum albumin (gm/dl)		3.70 (3.50–4.10)		3.70 (3.60–3.90)		3.70 (3.40–4.00)		0.297
Creatinine (mg/dl)		0.96 (0.80–1.10)		0.99 (0.90–1.02)		0.95 (0.80–1.10)		0.893
International normalized ratio		1.10 (1.04–1.24)		1.09 (1.00–1.20)		1.10 (1.00–1.20)		0.171
Alpha-fetoprotein (U/L)		257.00 (22.00–1210.00)		1031.00 (127.00–6418.00)		200.00 (13.30–1200.0)		0.269
HCC size		6.95 (5.00–10.00)		6.50 (5.00–9.30)		7.00 (5.00–12.00)		0.575
CHILD Score	A	601	96.6%	13	92.9%	67	95.7%	0.412
	B	21	3.4%	1	7.1%	3	4.3%	
Number of focal lesions	1	343	55.1%	9	64.3%	40	57.1%	0.699
	2	93	15.0%	1	7.1%	10	14.3%	
	3	28	4.5%	0	0.0%	6	8.6%	
	Multiple	158	25.4%	4	28.6%	14	20.0%	
HCC site	Right lobe	439	70.6%	12	85.7%	57	81.4%	0.100
	Left lobe	83	13.3%	0	0.0%	3	4.3%	
	Both	100	16.1%	2	14.3%	10	14.3%	
Portal vein status	Thrombosed	210	33.8%	4	28.6%	23	32.9%	0.954
	Patent	412	66.2%	10	71.4%	47	67.1%	
Malignant lymph node involvement		168	27.0%	4	28.6%	16	22.9%	0.781
Hepatic vein thrombosis		31	5.0%	0	0.0%	0	0.0%	0.127
Extra-hepatic spread		29	4.7%	3	21.4%	5	7.1%	0.026
BCLC	B	369	59.3%	7	50.0%	44	62.9%	0.651
	C	253	40.7%	7	50.0%	26	37.1%	
Previous treatment before Sorafenib		127	20.4%	3	21.4%	7	10.0%	0.088
Alive or dead	Alive	295	47.4%	3	21.4%	29	41.4%	0.107
	Dead	327	52.6%	11	78.6%	41	58.6%	
The Modified RECIST criteria after Sorafenib	Stable disease	198(31.8%)		4(28.6%		17(24.3%)		0.116
	Progressive disease	148(23.8%)		3(21.4%		10(14.3%)		
	Partial response	89(14.3%)		1(7.1%		10(14.3%)		
The Modified RECIST criteria after Regorafenib	Stable disease	25 (44.6%)		0 (0.0%)		2 (66.7%)		0.389
	Progressive disease	15(26.8%)		2 (66.7%)		1 (33.3%)		
	Partial response	16(28.6%)		1 (33.3%)		0 (0.0%)		
Sorafenib duration of treatment (days)		240.00 (90.00- 330.00)		225.00 (60.00–360.00)		270.00 (135.00–360.00)		0.498
Median (IQR) survival (days)		326.50 (151.00- 608.00)^a^		244.00 (105.00–387.00)^ab^		218.00 (111.00–454.00)^b^		0.021
Median (IQR) Time to progression (days)		180.00 (90.00- 300.00)		360.00 (60.00- 450.00)		195.00 (135.00–315.00		0.787

Variables carrying the same superscript letter are not statistically different.

We found no significant difference in the adverse events or the number of patients shifted to Regorafenib among the 3 groups (Supplementary table 4, 5). The median time to progression and the tumor response to treatment with Sorafenib and Regorafenib did not differ between the 3 groups. The median survival time was significantly lower in patients with non-viral-related HCC (218.00 (111.00–454.00)) than in patients with HCV-related HCC 326.50 (151.00- 608.00) *P*-value 0.048) (Table 6).

As we noticed that some patients could tolerate Sorafenib therapy for longer than 6 months while others did not, we divided our patients according to the duration of sorafenib treatment into those treated < 6 months and those treated > 6 months to study the characteristics of each group. Patients who continued treatment for less than 6 months (38.43%) showed higher baseline AFP, significantly lower number of patients with BCLC-B and more prevalent hepatic vein thrombosis, extra-hepatic spread and significantly worse survival (Table 7).

**Table 7:** Features of the studied patients according to the duration of Sorafenib therapy.

		Sorafenib therapy < 6months		Sorafenib therapy > 6months		*P* value
		Count	%	Count	%	
Age (years) (median (IQR)		63.00 (57.00–68.00)		62.00 (57.00–67.00)		0.089
Gender	Male	198	76.4%	316	76.1%	0.928
	Female	61	23.6%	99	23.9%	
Cigarette Smoking		39	15.1%	60	14.5%	0.830
Diabetes mellitus		64	24.7%	89	21.4%	0.071
Performance status	0	162	62.5%	306	73.7%	0.009
	1	76	29.3%	91	21.9%	
	2	16	6.2%	16	3.9%	
	3	5	1.9%	2	0.5%	
Liver status	Cirrhosis	256	98.8%	405	97.6%	0.389
	Non cirrhosis	3	1.2%	10	2.4%	
Hemoglobin (gm/dl)		12.40 (11.00–13.60)		12.50 (11.30–13.60)		0.213
Total leucocyte count (× 10^3^/ul)		6.10 (4.50–8.00)		5.90 (4.20–7.80)		0.598
Platelets (× 10^3^/ul)		173.00 (122.00–231.00)		160.00 (111.00–221.00)		0.020
Total bilirubin (mg/dl)		0.90 (0.70–1.20)		0.90 (0.70–1.10)		0.525
Alanine transferase (ALT) (U/L)		36.00 (25.00–52.00)		33.00 (23.00–47.00)		0.013
Aspartate transferase (AST) (U/L)		50.00 (34.00–72.00)		43.00 (30.00–66.00)		0.004
Serum albumin (gm/dl)		3.70 (3.40–4.02)		3.80 (3.50–4.10)		0.276
Creatinine (mg/dl)		1.00 (0.80–1.10)		0.91 (0.80–1.10)		0.573
International normalized ratio		1.10 (1.05–1.27)		1.10 (1.03–1.20)		0.284
Alpha-fetoprotein (U/L)		440.00 (33.80–2136.00)		200.00 (17.43–1200.00)		0.002
HCC size		7.00 (5.00–10.00)		6.80 (5.00–10.00)		0.576
CHILD Score	A	251	96.9%	403	97.1%	0.883
	B	8	3.1%	12	2.9%	
Number of focal lesions	1	132	51.0%	246	59.3%	< 0.001
	2	35	13.5%	67	16.1%	
	Multiple	83	35.5%	77	24.6%	
Portal vein status	Thrombosed	72	45.3%	83	43.7%	0.765
	Patent	87	54.7%	107	56.3%	
hepatic vein thrombosis	28	10.8%	3	0.7%	< 0.001	
Extra hepatic spread	18	6.9%	14	3.4%	0.034	
BCLC	B	137	52.9%	276	66.5%	< 0.001
	C	122	47.1%	139	33.5%	
Previous treatment before Sorafenib	62	23.9%	69	16.6%	0.020	
Sorafenib adverse events	Hypertension	43	16.6%	42	10.1%	0.014
	Hand and foot Syndrome	14	5.4%	16	3.9%	0.343
	Skin lesions	29	11.2%	63	15.2%	0.143
	Diarrhea	26	10.0%	28	6.7%	0.126
	Fatigue	75	29.0%	93	22.4%	0.056
	Abdominal pain	40	15.4%	32	7.7%	0.002
	Jaundice	98	37.8%	119	28.7%	0.013
	Elevated liver enzymes	63	24.3%	44	10.6%	< 0.001
	Ascites	33	12.7%	13	3.1%	< 0.001
	Anemia	37	14.3%	99	23.9%	0.003
	Hepatic encephalopathy	23	8.9%	3	0.7%	< 0.001
	Hematemesis	7	2.7%	5	1.2%	0.229
	Nausea/vomiting	10	3.9%	17	4.1%	0.880
Alive or dead	Alive	55	21.2%	263	63.4%	< 0.001
	Dead	204	78.8%	152	36.6%	
Performance status (after 1 month)	0	93	65.0%	295	77.0%	< 0.001
	1	36	25.2%	84	21.9%	
	2	8	5.6%	4	1.0%	
	3	6	4.2%	0	0.0%	
Performance status (3 months)	0	39	41.1%	272	79.5%	< 0.001
	1	44	46.3%	67	19.6%	
	2	11	11.6%	1	0.3%	
	3	1	1.1%	2	0.6%	
Shifted to Regorafenib		17	6.6%	50	12.0%	0.021
Follow up CT (mRECIST) after 3 months	Stable disease	48	41.4%	187	55.0%	< 0.001
	Progressive disease	66	56.9%	57	16.8%	
	Partial response	2	1.7%	96	28.2%	
Median (IQR) Overall Survival (days)		167.00 (104.00–349.00)		680.00(580.00- 743.00)		< 0.001

Patients who continued sorafenib for > 6 months were significantly less likely to develop hypertension, skin lesions, abdominal pain, jaundice, elevated liver enzymes, ascites, and anemia. On the other hand, they were more likely to develop hepatic encephalopathy. They had significantly better performance status after 1 and 3 months of treatment. They also showed better tumor response after 3 months of treatment (*P*-value < 0.001) with higher incidence of shifting to Regorafenib (Table 7).

## Discussion

Sorafenib is one of the HCC therapy guidelines either alone in the advanced stage or combined with loco-regional therapy in the intermediate stage after adequate multidisciplinary assessment. This study reported the characteristics, adverse events and outcome of patients with unresectable HCC treated with Sorafenib and Regorafenib and the impact of the underlying etiology of chronic liver disease.

We recruited our patients following the guidelines of the Egyptian National committee for control of viral hepatitis. The median age of our patients was 62 years with male predominance, 98% of our patients had cirrhosis, with the majority had Child–Pugh class A, 59.5% had BCLC stage B and the others had BCLC C stage, which was in harmony with previous studies^20,21^.

In our study, the median duration of treatment with Sorafenib was about 8 months. The most common causes for treatment discontinuation were Liver decompensation followed by patient demand. Jaundice was the most prevalent adverse event followed by fatigue, anemia, and elevated liver enzymes. A study by Raoul and colleagues, recruited 188 patients with Sorafenib-treated HCC with variable underlying etiologies (hepatitis C, alcoholic liver disease, NAFLD or undiagnosed), they had a median duration of therapy of 5.4 months. They reported the causes of sorafenib discontinuation as: another therapeutic option, major AEs or patient refusal, or no clear explanation. Their main reported AEs were skin toxicities (29%), elevated liver enzymes (22%), fatigue and weight loss (13%), diarrhea (10%), and hypertension (2%)^22^.

The response rate in our study was 14.2% while 32% of patients achieved stable disease and 22.8% had progressive disease. In a study done by Ferreira et al., the mean duration of sorafenib therapy was 9.7 months. 84% of their Brazilian patients received 800 mg per day, and the rest received half the dose. Their reported adverse events were diarrhea (33%), hand-foot syndrome (20.5%), mucositis (11.4%), fatigue and nausea (11.4%). They reported HCC progression in 48.4% of patients^21^.

On the other hand, Lee and his colleagues (2019) reported that of 222 Sorafenib-treated patients; eight (3.6%) achieved partial response (PR), 82 (36.9%) had stable disease (SD), 132 (59.5%) had progressive disease (PD) and none achieved complete response. Correlation between adverse events (AEs) and therapeutic responses revealed; hand-foot syndrome, hypertension, and diarrhea were (62.4%, 37.5%, and 25%) in the PR group, 40.8%, 13.4%, and 34.6% in the SD group and in PD group were (25%, 6.9%, and 15.9%)^23^.

In our study the median OS was 314 days (10.47 months (4.87–20.03)). The independent factors increasing mortality were baseline serum AST, size of HCC, hepatic vein thrombosis, the development of jaundice during treatment while shifting to Regorafenib was associated with lower risk of mortality. Similar to Rovesti et al.^24^, An independent predictor of a worse prognosis was AST. AST is often elevated by pathological processes that cause tissue damage, increased tumor cell turnover, and a greater proliferative state. We found no difference in survival between elderly and non-elderly patients similar to the retrospective study by Rovesti et al.^16^, and the prospective cohort study by Di Costanzo et al.^25^. This suggested that for senior people, sorafenib is deemed safe and effective.

The median OS of Sorafenib-treated patients reported in different studies ranges from 4 up to 17.4 months. It depends on the HCC stage, hepatic efficiency, performance status, and vascular invasion^21,22,26–28^. In addition, the reported predictors affecting OS among different studies are either *patient-related factors* (age, sex, race, PS, underlying liver disease etiology, albumin-bilirubin grade, neutrophil-to-lymphocyte ratio, Child–Pugh score, AFP, body mass index), *tumor-related factors* (number and size of the lesions, BCLC staging, extrahepatic spread, vascular invasion, and *sorafenib dosage*^21,22,26–28^. Our study reports higher survival time than previously reported in Egyptian patients by Nada et al.^26^ who recruited patients in 2015 (5 months) and Abdel-Rahman et al.^29^ who recruited patients in 2012 (6.25 months). This could be related to better patients’ selection and higher experience of the medical staff in patients’ management.

Regarding Regorafenib therapy, Bruix et al.^6^, proved that second line therapy with Regorafenib was associated with a significantly better OS, compared to placebo [10.6 versus 7.8 months, respectively]. A different phase II research conducted in 2013 by Bruix et al.^30^ with 36 patients with HCC shown good anticancer efficacy and accepted tolerability of Regorafenib, with a median OS of 13.8 months and a TTP of 4.3 months. In the Granito et al. study^31^, 216 Asian patients with Regorafenib-treated HCC achieved an OS of 10.6 months compared to 7.8 months with placebo with a 37% reduction in the risk of mortality and a 54% reduction in the risk of progression. They reported objective response in 11% of patients. Their reported AEs were hypertension (15%), hand-foot syndrome (13%), fatigue (9%), and diarrhea (3%). Regorafenib discontinuation was due to: elevated AST (2%), hand-foot syndrome (2%) and elevated ALT (1%).

We reported that OS was significantly lower in non-viral patients with sorafenib-treated HCC compared to viral-related HCC. Rovesti et al.^24^ reported that Sorafenib-treated patients with non-viral etiology had poorer OS than patients with viral etiologies. This could be attributed to non-screening of patients with non-viral chronic liver diseases, thus their HCC is diagnosed at an advanced stages. In addition, subgroup analysis of the SHARP and AP trials suggested a different sorafenib efficacy in patients with viral etiology. The mechanisms of action of Sorafenib, together with the diversity in tumor microenvironment, are capable of justifying the dissimilar antitumor profile. Bruix et al., reported a survival benefit favoring sorafenib over placebo was observed in patients with hepatitis C, low NLR and without extrahepatic spread^32^.

According to reports from 2008^33^, Egypt has the greatest HCV load in the world, with over 94% of patients having genotype 4^34,35^. Since the advent of direct-acting antiviral treatment (DAAs), the seroprevalence of HCV infection has decreased to 6.3%^5^ in 2015^36^, with an overall estimated 30% decrease in prevalence^37^. By 2018, nearly 2 million CHC patients got DAAs, accounting for 40% of the infected population, and SVR rates exceeded 90%^38^. This is why most patients in our study have HCV-related HCC. After the nationwide HBV vaccination in 1992, chronic hepatitis B, which was formerly Egypt’s second leading cause of HCC after HCV, dropped to third position.

NAFLD-related HCC was the most likely underlying etiology of the non-viral HCC in our patients. We excluded a number of underlying chronic liver diseases in our patients with nonviral HCC based on their medical history, lab test results, and clinical characteristics. These conditions included hemochromatosis, Wilson disease, primary sclerosing cholangitis, primary biliary cholangitis, and autoimmune liver disease. None of our patients had ever consumed alcohol since they were all pious Muslims.

In this study, Patients who continued treatment for less than 6 months (38.43%) showed higher baseline AFP, less patients with BCLC-C than B and more prevalent hepatic vein thrombosis, extra-hepatic spread and with significantly worse OS. Chan et al.^39^ reported that 15.49% of their patients could tolerate Sorafenib therapy for at least 6 months, decreasing to only 4.9% with sorafenib treatment > 12 months. They mentioned that it could be attributed to Sorafenib dose, performance status and the HCC tumor characteristics. It was reported that Sorafenib is only beneficial for about 30% of patients, but within 6 months, most of these individuals develop drug resistance^40^. For this reason, we decided to use the 6-month period to split our patients based on how long they had been receiving sorafinib medication. Therefore, it is essential to carefully assess each patient’s features and the events that have occurred during their sorafenib treatment in order to determine which individuals are best suited to continue on sorafenib and which ones should undergo Regorafenib as a second-line treatment.

Limitations of the study include its retrospective design. The number of patients with HCV-related HCC markedly exceeds the 2 other etiological groups but this reflects the true percentage of each etiology in the Egyptian patients with HCC. In addition, limited Regorafenib subgroup analysis (small in number) weaken generalizability and Only 68% of patients had evaluable mRECIST criteria as some patients lost follow-up before they perform the first on-treatment imaging modality either due to patients’ death, unwillingness to continue therapy or patient absence and could not be reached. Our study’s key advantages are its considerable sample size and patient participation across multiple centers. We examined medical data that was gathered in real-world settings, simulating typical medical care and offering significant external validity.

## Supplementary Information

Supplementary Information.

## Data Availability

The datasets used and/or analysed during the current study available from the corresponding author on reasonable request.
