# Supplementary material for: Tyrosine kinase inhibitors were well-tolerated among patients with different etiologies of advanced HCC with lower survival in non-viral patients
Source: Sci Rep. 2025 Jun 27;15:20323. doi: 10.1038/s41598-025-05828-x (PMC12205039; doi:10.1038/s41598-025-05828-x)
Supplement: Supplementary file 1 — Supplementary Information. [file 41598_2025_5828_MOESM1_ESM.docx]

**Supplementary Table 1: Laboratory changes during sorafenib and Regorafenib treatment**

|  | **Median** | **1^st^ quartile** | **3^rd^ quartile** |
| --- | --- | --- | --- |
| **Hemoglobin (1 month)** | 12.60 | 11.10 | 14.00 |
| **TLC (1 month)** | 5.50 | 4.00 | 7.30 |
| **PLT (1 month)** | 150.00 | 110.00 | 213.00 |
| **ALT (1 month)** | 38.00 | 25.00 | 53.00 |
| **AST (1 month)** | 50.00 | 36.00 | 74.00 |
| **Total BILIRUBIN (1 month)** | 1.10 | 0.80 | 1.50 |
| **creatinine after (1 month)** | 0.90 | 0.74 | 1.00 |
| **albumin (1 month)** | 3.60 | 3.20 | 3.90 |
| **AFP (1 month)** | 188.50 | 11.30 | 1198.00 |
| **INR (1 month)** | 1.20 | 1.10 | 1.30 |
| **Hemoglobin (3 months)** | 12.30 | 11.00 | 13.50 |
| **TLC (3 months)** | 5.20 | 4.00 | 6.80 |
| **PLT (3 months)** | 143.00 | 105.00 | 208.00 |
| **ALT (3 months)** | 39.00 | 26.00 | 53.00 |
| **AST (3 months)** | 49.00 | 35.00 | 70.00 |
| **Total BILIRUBIN (3 months)** | 1.10 | 0.90 | 1.50 |
| **creat (3 months)** | 0.90 | 0.80 | 1.02 |
| **albumin (3 months)** | 3.60 | 3.20 | 4.00 |
| **AFP (3 months)** | 180.00 | 12.00 | 950.00 |
| **INR (3 months)** | 1.20 | 1.05 | 1.30 |
| **hemoglobin (6 months)** | 12.00 | 11.00 | 13.50 |
| **TLC (6 months)** | 5.20 | 4.00 | 7.00 |
| **PLT (6 months)** | 141.50 | 98.00 | 203.00 |
| **ALT (6 months)** | 39.00 | 27.00 | 52.00 |
| **AST (6 months)** | 45.00 | 34.00 | 65.00 |
| **Total BILIRUBIN (6 months)** | 1.15 | 0.90 | 1.50 |
| **creat (6 months)** | 0.95 | 0.80 | 1.00 |
| **albumin (6 months)** | 3.60 | 3.30 | 4.00 |
| **AFP (6 months)** | 100.00 | 9.30 | 700.00 |
| **INR (6 months)** | 1.14 | 1.00 | 1.25 |
| **Hemoglobin (9 months)** | 11.90 | 11.00 | 12.50 |
| **WBCs (9 months)** | 5.00 | 4.00 | 6.30 |
| **PLT (9 months)** | 135.00 | 99.00 | 183.00 |
| **ALT (9 months)** | 38.00 | 26.00 | 49.00 |
| **AST (9 months)** | 46.00 | 37.00 | 56.00 |
| **Total BILIRUBIN (9 months)** | 1.10 | 0.90 | 1.40 |
| **creat (9 months)** | 1.00 | 0.90 | 1.00 |
| **albumin (9 months)** | 3.70 | 3.30 | 4.00 |
| **AFP (9 months)** | 67.00 | 6.30 | 450.00 |
| **INR (9 months)** | 1.10 | 1.00 | 1.30 |
| **Hemoglobin (12 months)** | 11.30 | 10.60 | 12.50 |
| **WBCs (12 months)** | 5.00 | 4.00 | 6.30 |
| **platelets (12 months)** | 147.00 | 100.00 | 188.00 |
| **ALT (12 months)** | 39.00 | 26.00 | 51.00 |
| **AST (12 months)** | 50.00 | 38.00 | 56.00 |
| **Total BILIRUBIN (12 months)** | 1.00 | 0.90 | 1.30 |
| **creat (12 months)** | 1.00 | 0.80 | 1.00 |
| **albumin (12 months)** | 3.70 | 3.30 | 4.00 |
| **AFP (12 months)** | 29.93 | 8.25 | 380.00 |
| **INR (12 months)** | 1.10 | 1.00 | 1.30 |
| **HB (1 month after Regorafenib)** | 12.20 | 11.00 | 13.50 |
| **TLC (1 month after Regorafenib)** | 5.50 | 3.80 | 7.00 |
| **PLT (1 month after Regorafenib)** | 148.00 | 125.00 | 202.00 |
| **ALT (1 month after Regorafenib)** | 37.00 | 24.00 | 53.00 |
| **AST (1 month after Regorafenib)** | 40.00 | 33.00 | 55.00 |
| **Total BILIRUBIN (1 month after Regorafenib)** | 1.10 | 0.80 | 1.20 |
| **creat (1 month after Regorafenib)** | 0.90 | 0.80 | 1.00 |
| **albumin (1 month after Regorafenib)** | 3.75 | 3.40 | 4.00 |
| **AFP (1 month after Regorafenib)** | 150.00 | 11.00 | 1200.00 |
| **INR (1 month after Regorafenib)** | 1.20 | 1.10 | 1.30 |
| **Hemoglobin (3 months after Regorafenib)** | 12.00 | 11.00 | 12.50 |
| **TLC (3 months after Regorafenib)** | 6.00 | 4.60 | 7.00 |
| **PLT (3 months after Regorafenib)** | 141.00 | 120.00 | 198.00 |
| **ALT (3 months after Regorafenib)** | 39.00 | 25.00 | 52.00 |
| **AST (3 months after Regorafenib)** | 44.00 | 34.00 | 54.00 |
| **Total BILIRUBIN (3 months after Regorafenib)** | 1.10 | 1.00 | 1.40 |
| **creat (3 months after Regorafenib)** | 1.00 | 0.90 | 1.00 |
| **albumin (3 months after Regorafenib)** | 3.70 | 3.30 | 4.00 |
| **AFP (3 months after Regorafenib)** | 149.00 | 16.50 | 560.00 |
| **INR (3 months after Regorafenib)** | 1.10 | 1.00 | 1.30 |

**Supplementary Table 2: ECOG performance status changes during sorafenib treatment**

|  | | Count | Percent |
| --- | --- | --- | --- |
| **performance status (1 month)** | **0** | 393 | 73.6% |
|  | **1** | 122 | 22.8% |
|  | **2** | 13 | 2.4% |
|  | **3** | 6 | 1.1% |
| **performance status (3 months)** | **0** | 315 | 71.3% |
|  | **1** | 112 | 25.3% |
|  | **2** | 12 | 2.7% |
|  | **3** | 3 | 0.7% |
| **performance status at 6 months** | **0** | 208 | 73.5% |
|  | **1** | 62 | 21.9% |
|  | **2** | 10 | 3.5% |
|  | **3** | 3 | 1.1% |
| **performance status at 9 months** | **0** | 117 | 74.1% |
|  | **1** | 30 | 19.0% |
|  | **2** | 9 | 5.7% |
|  | **3** | 2 | 1.3% |
| **performance status at 12 months** | **0** | 48 | 77.4% |
|  | **1** | 11 | 17.7% |
|  | **2** | 3 | 4.8% |

**Supplementary Table 3: Correlation between the duration of Sorafenib therapy and baseline variables**

| **Variable** | **Duration of treatment (days)** | |
| --- | --- | --- |
|  | **Correlation Coefficient** | **P value** |
| **Age (years)** | -0.085- | 0.112 |
| **Hemoglobin** | 0.085 | 0.113 |
| **White blood cells** | 0.005 | 0.928 |
| **Platelets** | -0.119- | 0.026 |
| **Total bilirubin** | -0.042- | 0.435 |
| **ALT** | -0.052- | 0.330 |
| **AST** | -0.101- | 0.060 |
| **Albumin** | -0.076- | 0.155 |
| **Creatinine** | 0.008 | 0.881 |
| **INR** | 0.011 | 0.843 |
| **AFP** | -0.202- | < 0.001 |
| **F.L size or size of largest lesion if 2 or multiple** | 0.026 | 0.639 |
| **Survival time (days)** | 0.732 | < 0.001 |

**Supplementary table 4: Comparison between baseline variables and before treatment stop**

|  | **Baseline variables** | | **Variables before treatment stop** | | **P-value** |
| --- | --- | --- | --- | --- | --- |
|  | **Median** | **IQR** | **Median** | **IQR** |  |
| **Hemoglobin (gm/dl)** | 12.50 | 11.20-13.60 | 11.80 | 10.80-13.00 | <0.001 |
| **Total leucocyte count (x10^3^/ul)** | 6.00 | 4.35-7.80 | 5.40 | 4.00-7.20 | <0.001 |
| **Platelets** | 165.00 | 116.00-224.50 | 150.00 | 110.00-211.50 | 0.021 |
| **Total bilirubin (mg/dl)** | 0.90 | 0.70-1.20 | 1.20 | 0.90-1.74 | <0.001 |
| **Alanine transferase (ALT) (U/L)** | 34.00 | 24.00-49.00 | 40.00 | 26.00-55.00 | <0.001 |
| **Aspartate transferase (AST) (U/L)** | 45.00 | 32.00-70.00 | 54.00 | 38.00-78.50 | <0.001 |
| **Serum albumin (gm/dl)** | 3.70 | 3.50-4.01 | 3.50 | 3.00-3.80 | <0.001 |
| **Creatinine (mg/dl)** | 0.95 | 0.80-1.10 | 0.90 | 0.80-1.09 | 0.189 |
| **International normalized ratio** | 1.10 | 1.04-1.23 | 1.20 | 1.06-1.30 | <0.001 |
| **Alpha-fetoprotein (U/L)** | 400.00 | 21.85-1266.00 | 504.50 | 34.50-1200.00 | 0.114 |

**Supplementary table 5: Adverse events affecting the studied patients according to their underlying etiology**

|  | **Chronic hepatitis C**  **N=622** | | **Chronic hepatitis B**  **N=14** | | **Non-viral**  **N= 70** | | **P value** |
| --- | --- | --- | --- | --- | --- | --- | --- |
|  | **Count** | **%** | **Count** | **%** | **Count** | **%** |  |
| **Hypertension** | 75 | 12.1% | 1 | 7.1% | 11 | 15.7% | 0.634 |
| **hand and foot syndrome** | 30 | 4.8% | 0 | 0.0% | 1 | 1.4% | 0.495 |
| **skin lesions** | 80 | 12.9% | 3 | 21.4% | 9 | 12.9% | 0.615 |
| **Diarrhea** | 49 | 7.9% | 3 | 21.4% | 4 | 5.7% | 0.140 |
| **fatigue** | 154 | 24.8% | 6 | 42.9% | 14 | 20.0% | 0.189 |
| **abdominal pain** | 68 | 10.9% | 2 | 14.3% | 6 | 8.6% | 0.713 |
| **Jaundice** | 193 | 31.0% | 2 | 14.3% | 23 | 32.9% | 0.406 |
| **Elevated liver enzymes** | 97 | 15.6% | 2 | 14.3% | 10 | 14.3% | 0.960 |
| **Hematemesis** | 13 | 2.1% | 0 | 0.0% | 1 | 1.4% | 1 |
| **nausea/vomiting** | 25 | 4.0% | 1 | 7.1% | 1 | 1.4% | 0.365 |
| **shifted to Regorafenib** | 62 | 10.0% | 3 | 21.4% | 3 | 4.3% | 0.089 |

**Supplementary table 6: Difference in duration of therapy, OS, TTP and shifting to Regorafenib among patients with HCV-related and non-viral HCC**

|  | **HCV-related HCC** | | | **Non-viral HCC** | | |  |
| --- | --- | --- | --- | --- | --- | --- | --- |
|  | **Median** | **1^st^ quartile** | **3^rd^ quartile** | **Median** | **1^st^ quartile** | **3^rd^ quartile** | **P value** |
| **duration of treatment (days)** | 240.00 | 90.00 | 330.00 | 270.00 | 135.00 | 360.00 | 0.236 |
| **survival duration (days)** | 326.50 | 151.00 | 608.00 | 218.00 | 111.00 | 454.00 | 0.016 |
| **time to progression (days)** | 180.00 | 90.00 | 300.00 | 195.00 | 135.00 | 315.00 | 0.941 |

|  | **HCV** | | **nonviral** | |  |
| --- | --- | --- | --- | --- | --- |
|  | **Count** | **%** | **Count** | **%** | **P value** |
| **shifted to Regorafenib** | 62 | 10.0% | 3 | 4.3% | 0.122 |

Supplementary figure 1: Kaplan Meier for survival stratified by etiology


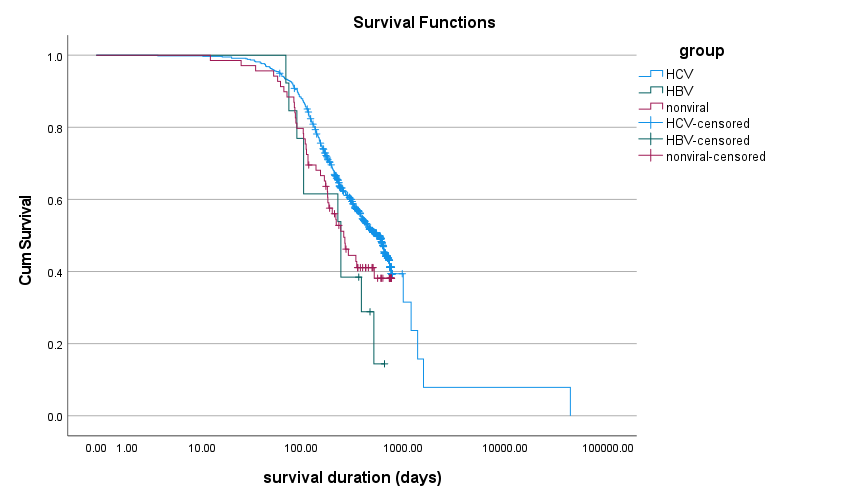


| **Means and Medians for Survival Time** | | | | | | | | |
| --- | --- | --- | --- | --- | --- | --- | --- | --- |
| **group** | **Mean^a^** | | | | **Median** | | | |
|  | **Estimate** | **Std. Error** | **95% Confidence Interval** | | **Estimate** | **Std. Error** | **95% Confidence Interval** | |
|  |  |  | **Lower Bound** | **Upper Bound** |  |  | **Lower Bound** | **Upper Bound** |
| **HCV** | 3938.748 | 2937.725 | 0.000 | 9696.689 | 552.000 | 58.804 | 436.744 | 667.256 |
| **HBV** | 294.692 | 58.986 | 179.079 | 410.306 | 244.000 | 81.274 | 84.703 | 403.297 |
| **Non-viral** | 394.693 | 37.596 | 321.004 | 468.382 | 260.000 | 46.948 | 167.982 | 352.018 |
| **Overall** | 3867.586 | 2884.330 | 0.000 | 9520.872 | 489.000 | 53.672 | 383.803 | 594.197 |
| a. Estimation is limited to the largest survival time if it is censored. | | | | | | | | |

| **Overall Comparisons** | | | |
| --- | --- | --- | --- |
|  | **Chi-Square** | **df** | **P value** |
| **Log Rank (Mantel-Cox)** | 7.395 | 2 | 0.025 |
| Test of equality of survival distributions for the different levels of group. | | | |

Kaplan Meier for survival stratified by **BCLC stage**


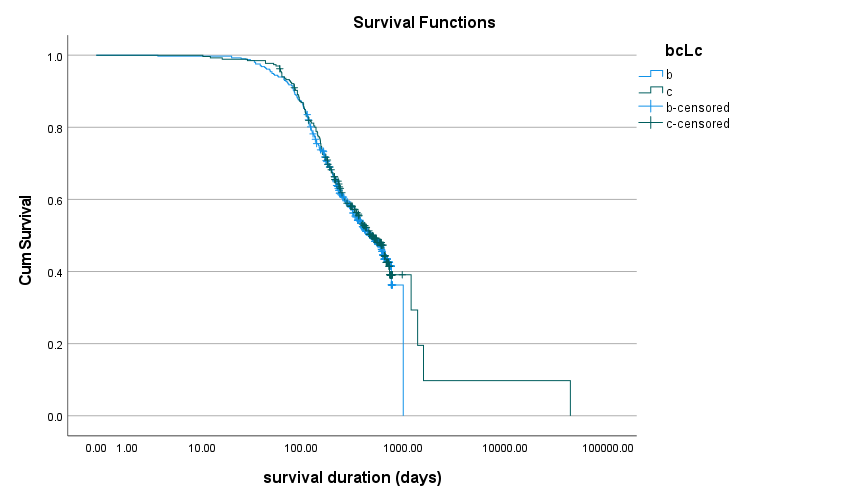


| **Means and Medians for Survival Time** | | | | | | | | |
| --- | --- | --- | --- | --- | --- | --- | --- | --- |
| **bcLc** | **Mean^a^** | | | | **Median** | | | |
|  | **Estimate** | **Std. Error** | **95% Confidence Interval** | | **Estimate** | **Std. Error** | **95% Confidence Interval** | |
|  |  |  | **Lower Bound** | **Upper Bound** |  |  | **Lower Bound** | **Upper Bound** |
| **b** | 541.843 | 23.152 | 496.466 | 587.220 | 489.000 | 69.113 | 353.538 | 624.462 |
| **c** | 4741.630 | 3536.514 | 0.000 | 11673.198 | 485.000 | 84.442 | 319.494 | 650.506 |
| **Overall** | 3867.586 | 2884.330 | 0.000 | 9520.872 | 489.000 | 53.672 | 383.803 | 594.197 |
| a. Estimation is limited to the largest survival time if it is censored. | | | | | | | | |

| **Overall Comparisons** | | | |
| --- | --- | --- | --- |
|  | **Chi-Square** | **df** | **P value** |
| **Log Rank (Mantel-Cox)** | 0.058 | 1 | 0.809 |
| Test of equality of survival distributions for the different levels of bcLc. | | | |

Kaplan Meier for **TTP** stratified by etiology


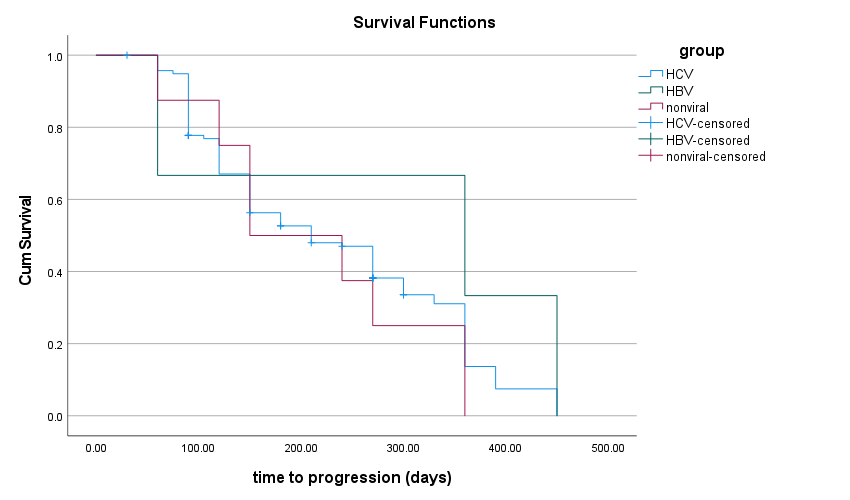


| **Means and Medians for Survival Time** | | | | | | | | |
| --- | --- | --- | --- | --- | --- | --- | --- | --- |
| **group** | **Mean^a^** | | | | **Median** | | | |
|  | **Estimate** | **Std. Error** | **95% Confidence Interval** | | **Estimate** | **Std. Error** | **95% Confidence Interval** | |
|  |  |  | **Lower Bound** | **Upper Bound** |  |  | **Lower Bound** | **Upper Bound** |
| **HCV** | 232.551 | 11.950 | 209.130 | 255.973 | 210.000 | 31.280 | 148.691 | 271.309 |
| **HBV** | 290.000 | 117.898 | 58.919 | 521.081 | 360.000 | 244.949 | 0.000 | 840.100 |
| **nonviral** | 213.750 | 39.458 | 136.413 | 291.087 | 150.000 | 56.569 | 39.126 | 260.874 |
| **Overall** | 232.773 | 11.421 | 210.387 | 255.159 | 210.000 | 29.802 | 151.588 | 268.412 |
| a. Estimation is limited to the largest survival time if it is censored. | | | | | | | | |

| **Overall Comparisons** | | | |
| --- | --- | --- | --- |
|  | **Chi-Square** | **df** | **P value** |
| **Log Rank (Mantel-Cox)** | 1.685 | 2 | 0.431 |
| Test of equality of survival distributions for the different levels of group. | | | |

Kaplan Meier for **TTP** stratified by **BCLC stage**


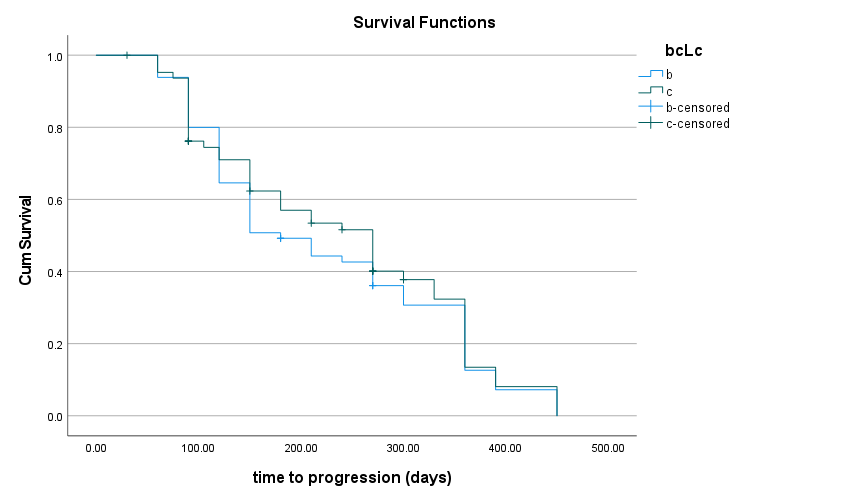


| **Means and Medians for Survival Time** | | | | | | | | |
| --- | --- | --- | --- | --- | --- | --- | --- | --- |
| **bcLc** | **Mean^a^** | | | | **Median** | | | |
|  | **Estimate** | **Std. Error** | **95% Confidence Interval** | | **Estimate** | **Std. Error** | **95% Confidence Interval** | |
|  |  |  | **Lower Bound** | **Upper Bound** |  |  | **Lower Bound** | **Upper Bound** |
| **b** | 224.997 | 15.732 | 194.162 | 255.832 | 180.000 | 27.482 | 126.136 | 233.864 |
| **c** | 241.516 | 16.640 | 208.900 | 274.131 | 270.000 | 34.906 | 201.584 | 338.416 |
| **Overall** | 232.773 | 11.421 | 210.387 | 255.159 | 210.000 | 29.802 | 151.588 | 268.412 |
| a. Estimation is limited to the largest survival time if it is censored. | | | | | | | | |

| **Overall Comparisons** | | | |
| --- | --- | --- | --- |
|  | **Chi-Square** | **df** | **P value** |
| **Log Rank (Mantel-Cox)** | 0.237 | 1 | 0.626 |
| Test of equality of survival distributions for the different levels of bcLc. | | | |
